# Axial bonds at the T1 Cu site of *Thermus thermophilus* SG0.5JP17‐16 laccase influence enzymatic properties

**DOI:** 10.1002/2211-5463.12633

**Published:** 2019-04-09

**Authors:** Yanyun Zhu, Yi Zhang, Jiangbo Zhan, Ying Lin, Xiaorong Yang

**Affiliations:** ^1^ Guangdong Provincial Key Laboratory of Fermentation and Enzyme Engineering School of Biology and Biological Engineering South China University of Technology Guangzhou China; ^2^ Guangdong Research Center of Industrial Enzyme and Green Manufacturing Technology School of Biology and Biological Engineering South China University of Technology Guangzhou China

**Keywords:** axial bond, copper, kinetic analysis, laccase, redox potential, site‐directed mutation

## Abstract

Laccase is a multi‐copper oxidase which oxidizes substrate at the type 1 copper site, simultaneously coupling the reduction of dioxygen to water at the trinuclear copper center. In this study, we used site‐directed mutagenesis to study the effect of axial bonds between the metal and amino acid residue side chains in lacTT. Our kinetic and spectral data showed that the replacement of the axial residue with non‐coordinating residues resulted in higher efficiency (*k*_cat_/*K*_m_) and a lower Cu^2+^ population at the type 1 copper site, while substitution with strongly coordinating residues resulted in lower efficiency and a higher Cu^2+^ population, as compared with the wild‐type. The redox potentials of mutants with hydrophobic axial residues (Ala and Phe) were higher than that of the wild‐type. In conclusion, these insights into the catalytic mechanism of laccase may be of use in protein engineering to fine‐tune its enzymatic properties for industrial application.

**Abbreviations**
lacTT: *Thermus thermophilus* SG0.5JP17‐16 laccase
MCO: multicopper oxidase
T1 Cu: type 1 copper
T2 Cu: type 2 copper
T3 Cu: type 3 copper
TNC: trinuclear copper cluster

Laccases (benzenediol: oxygen oxidoreductase; EC1.10.3.2) are copper‐containing polyphenol oxidases, belong to the multicopper oxidase (MCO) family including ceruloplasmin and ascorbate oxidase 1, 2. They can catalyze the oxidation of a variety of substrates, such as phenolic compounds, non‐phenolic compounds and aromatic amines, extensively used in the conversion of plant biomass, and decolorization and detoxification of textile wastewater 3, 4.

The active site of laccase contains four Cu ions, which can be divided into three types according to their spectroscopic and magnetic characteristics, namely type 1 copper (T1 Cu), type 2 copper (T2 Cu) and type 3 copper (T3 Cu) 5. The T1 Cu is coordinated by two histidines, one cysteine and an axial ligand, has an intense absorption band at around 600 nm in the UV–visible spectrum and a small parallel hyperfine coupling constant in EPR (A|| = (43–90) × 10^−4 ^cm^−1^), both resulting from the covalent bond between Cu and S(Cys), which generates a ligand‐to‐metal (S_Cys_π → Cu) charge transition 6. The T2 Cu is coordinated by two histidines and a water molecule. There is no absorption band in the UV–visible spectrum but a parallel hyperfine coupling is recorded in EPR (A|| = (105–201) × 10^−4 ^cm^−1^) 6, 7. The T3 Cu contains two copper atoms, namely T3α Cu and T3β Cu, each of which is coordinated by three histidine residues. The T3 Cu is characterized by a broad absorption band at 330 nm resulting from the hydroxyl bridge‐to‐metal (HO^−^–Cu) charge transition, but lacks EPR signal due to the anti‐ferromagnetic coupling of two copper ions 7. The T2 Cu and two T3 Cu are described as the trinuclear copper cluster (TNC) 8.

The catalytic reaction of laccase is a complex process. It generally includes the following steps: oxidation of the substrate, electron transfer inside the molecule, and reduction of oxygen molecules to water. Substrate oxidation occurs near the T1 Cu, and electrons are transferred through the protein via the Cys–His–N(His) pathway to the TNC, where dioxygen reduction occurs 7.

The activity and the whole reaction characteristics of laccase are closely related to the level of redox potential (*E*°) 9. The T1 Cu^2+^ ion is the electron acceptor of reducing substrate, which determines the reduction potential of laccase. The enzymatic reaction requires that the reduction potential of the substrate must be lower than or slightly higher than that of the T1 Cu site 10. The axial ligand of the T1 Cu site is connected to the redox potential of laccase 11. Table 1 shows the redox potential of several typical blue‐copper proteins and their corresponding axial coordination amino acids 10, 12, 13, 14, 15, 16, 17, 18, 19. The difference in the coordination environment leads to different redox potentials. In the fungal laccases, the axial ligand of T1 Cu is a hydrophobic amino acid, phenylalanine or leucine 7, 16, which have a high redox potential of up to 790 mV. For the blue‐copper protein with a methionine as the axial ligand, the redox potential is relatively low, down to 310 mV 18. The non‐coordinating residue, phenylalanine or leucine, at the axial position may be an important determinant for the high redox potential of fungal laccase 16, 20.

**Table 1:** Redox potentials of the T1 Cu site for oxidases from different sources

Source	Species	Enzyme	Axial ligand	*E* ^o^ (mv)
*Trametes versicolor*	Fungus	Laccase	Phe	790 12, 13
*Trametes hirsuta*	Fungus	Laccase	Phe	760 14
*Coprinopsis cinerea*	Fungus	Laccase	Leu	550 15
*Melanocarpus *albomyces	Fungus	Laccase	Leu	470 10
*Bacillus subtilis*	Bacterium	Laccase	Met	455 16
*Pseudomonas aeruginosa*	Bacterium	Azurin	Met	310 17
*Toxicodendron vernicifluum*	Plant	Laccase	Met	430 18
*Cucurbita pepo* L.	Plant	Ascorbic oxidase	Met	340 10
Homo sapiens	Mammal	Ceruloplasmin	Met	490 19

*Thermus thermophilus* SG0.5JP17‐16 laccase (lacTT) has beneficial characteristics: it is thermostable, has a high tolerance for chloride 21, and has a very high decolorization efficiency for some industrial dyes: over 90% for azo dyes, congo red, reactive black B, and reactive black WNN; 73% for anthraquinone dyes and remazol brilliant blue R after 24 h 21. Thus, the lacTT is a good candidate to decolor and detoxify textile wastewater. We have identified four key amino acid residues affecting the catalytic reaction of the lacTT, which are involved in substrate binding, proton transfer and the reduction of O_2_ 22. The lacTT has typical structural features of multiple copper oxidases (Fig. 1A) 16. The T1 Cu is coordinated by His397, His455, Cys450 and the axial ligand, Met460 (Fig. 1B). In this study, we took the axial ligand, Met460, as the target to look for mutants with enhanced catalytic efficiency. The Met460 residue was mutated to non‐coordinating amino acid residues (alanine, phenylalanine or leucine), or strongly coordinating residues (histidine or glutamine) by site‐directed mutagenesis to comprehensively explore the effect of the type of axial ligand on the catalytic reaction. How the mutations would affect the enzymatic properties *k*_cat_, *K*_m_ and spectral properties was examined. How the nature of the axial residue of the T1 Cu ion influenced the structure and *E*° of this copper site in the lacTT was analyzed. These results might contribute to the understanding of the basic mechanism by which the laccase catalyzes the oxidation of the substrate, and further provide a new strategy to engineer the enzyme for industrial application.

**Figure 1:**
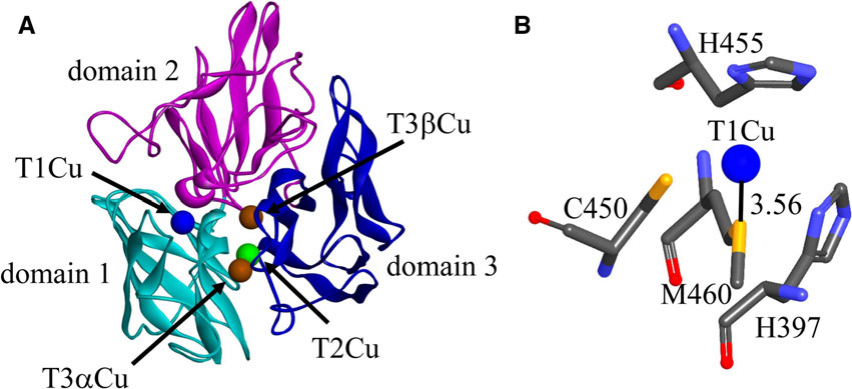
(A) The 3D structure model of lacTT containing three domains and four Cu atoms. (B) The coordination environment of the T1 Cu site. The T1 Cu atom is coordinated by the C460, H455 and C450 ligands; at its axial position, the fourth ligand, M460, weakly interacts with the Cu atom, forming the S_M__et_–Cu bond.

## Materials and methods

### Preparation of lacTT mutants

The method of constructing lacTT mutants is similar to the previous report 22. premier 5.0 (Premier Biosoft International, Palo Alto, CA, USA) was used to design the site‐directed mutagenesis primers, and the mutant genes were obtained by overlap‐extension PCR using the pET30a‐lacTT recombinant plasmid as template. The lacTT and mutated genes were cloned into the plasmids pET‐30a(+) that were digested with NcoI and HindIII. The lacTT gene was synthesized by Nanjing Kingsley Biotechnology Co. Ltd, Nanjing, China. The plasmid pET‐30a(+) and the cloning host *Escherichia coli* Top10 were purchased from Novagen, Madison, WI, USA.

### Gene expression and protein purification

Wild‐type and mutant proteins were recombinantly expressed in *E. coli* BL21(DE3) as described in Liu *et al*. 22. The details were as follows. Firstly, recombinant plasmids were transferred into *E. coli* BL21 competent cells. The expression host with recombinant plasmids was cultured in LB medium at 37 °C and 200 r.p.m. until the *D*_600_ value reached 0.6–1. Then, IPTG (0.1 mm) was added into the medium to induce expression overnight.

After the fermentation was completed, the bacterial cells were harvested by centrifugation and crushed by ultrasonics to obtain crude protein extract. Ni^2+^ affinity chromatography was used for protein purification. The chromatography system used in this study was an ÄKTA purifier, FPLC, and the Ni^2+^ affinity column was the HisTrap™ FF (GE Healthcare, Piscataway, NJ, USA). The purity of protein samples was analyzed by SDS/PAGE. Protein concentration was determined by the Bradford method; a protein standard curve was calculated using BSA as a standard protein.

### Enzyme activity and kinetics

The laccase activity was determined by monitoring the oxidation of guaiacol. The reaction system contained 0.0566 μm laccase or mutants, 2 mm guaiacol, 10 mm CuSO_4_ and 100 mm sodium phosphate buffer (pH 6.0). The reaction was performed for 5 min in a water bath of 90 °C, with an ice bath to cool the sample to room temperature, and was followed at 465 nm (ε = 12 100 m ^−1^·cm^−1^) with a MultiSkan Ascent 354 spectrophotometer from Thermo Industries (Thermo Fisher Scientific, Kanagawa, Japan). The reaction rate was calculated by the Lambert–Beer law, and was measured as the function of temperature of 60–95 °C at the optimum pH, pH 4.5–8.0 at 90 °C, and time of 0–4 h. The highest enzymatic activity was set to 100%. Initial reaction rate *V*_max_ and the Michaelis constant *K*_m_ were obtained using the Lineweaver–Burk plot in the concentration range of 62.5–4000 μm guaiacol, and the formula (*k*_cat_ = *V*_max_/[*E*_0_]) was used to calculate turnover number (*k*_cat_) and enzyme catalytic efficiency (*k*_cat_/*K*_m_). All experiments were carried out in triplicate.

### UV–visible spectroscopy

The mixture contained 56.6 μm (3 mg·mL^−1^) wild‐type or mutant laccase and 10 mm sodium phosphate buffer, pH 6.0. UV–visible absorption spectra were acquired from 300 to 800 nm by a Hitachi U‐3010 UV‐Vis spectrophotometer (Hitachi, Tokyo, Japan).

### Redox potential

The redox potential of T1 Cu in laccase was determined by the previously reported method 23. The *E* ^o^ of the T1 Cu center was measured by protein redox titration with K_3_Fe(CN)_6_/K_4_Fe(CN)_6_ as the medium at 25 °C. The reaction mixture contained 56.6 μm enzyme, 10 mm CuSO_4_, 0–10 mm K_3_Fe(CN)_6_, 5–15 mm K_3_Fe(CN)_6_ and 100 mm sodium phosphate buffer (pH 6.0). The absorbance of laccase in the range of 550–800 nm was monitored by adding redox couples with different ratios in the reaction system until the absorption peak at around 608 nm disappeared, indicating that the reaction had reached equilibrium. The whole measurement process was carried out under anaerobic conditions. The redox potential was calculated by the Nernst equation.

### Structural analysis

In the previous work, we have obtained a three‐dimensional (3D) structural model of lacTT 22. Starting from the known 3D model of wild‐type laccase, we used discovery studio 3.5 (BIOVIA, San Diego, CA, USA) (Build Mutants module) to perform virtual amino acid mutations of M460A, M460F, M460H, M460L and M460Q laccases.

## Results

### Thermal characterization of wild‐type and mutant laccases

**Figure 2:**
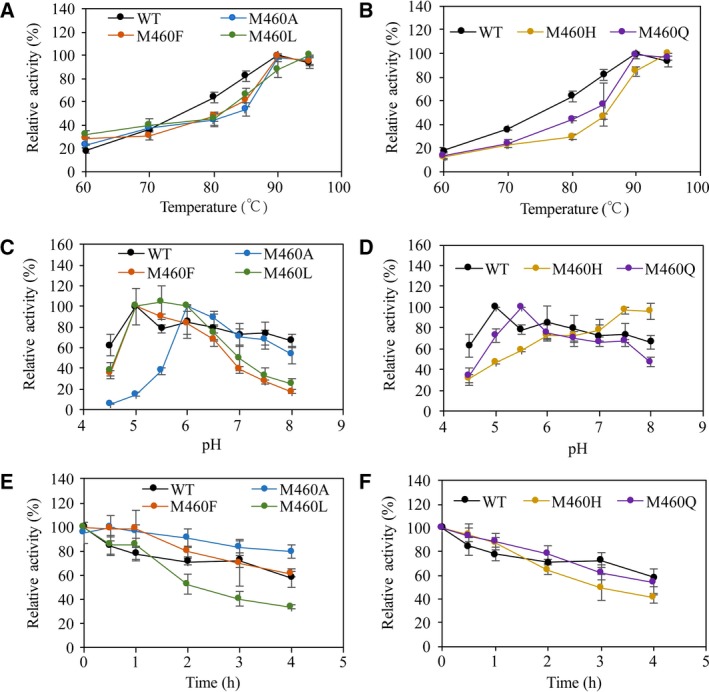
(A, B) Effect of temperature on the activity of the WT lacTT and mutants M460L, M460F and M460A (A), and M460H and M460Q (B). (C, D) Thermostability curves of the WT lacTT and mutants at 80 °C, pH 6.0. The residual activity was determined after incubation for 0–4 h at 90 °C. Laccase activity was normalized to the optimum activity value. (E, F) Effect of pH on the activity of wild‐type lacTT and mutants. Activities were measured in 0.1 m Na_2_ HPO_4_/KH_2_ PO_4_ buffer (pH 6.0–8.0) with 2 mm guaiacol as substrate. The standard deviation of each point is shown by error bars and was determined by three independent experiments.

The enzymatic activity was measured in the temperature range 60–95 °C. Optimal reaction temperatures of wild‐type and mutant enzymes were determined at pH 6 (Fig. 2A,B). The M460A, M460F and M460Q mutants and wild‐type lacTT exhibited a similar optimal temperature of 90 °C. The optimal temperature of the M460L and M460H mutants was 95 °C, slightly higher than that of the wild‐type laccase. The enzymatic activities of lacTT and mutants were recorded as a function of pH from 4.5 to 8.0 at 90 °C (Fig. 2C,D). The optimal pH value of the M460A mutant was similar to that of the wild‐type, 6.0. The optimal pH values of the M460L, M460F and M460Q mutants moved to acidic value by 0.5–1 units. The mutant M460H had the optimal pH value of 7.5, which shifted toward the alkaline. It was speculated that mutations might affect the gain and loss of electrons at the T1 Cu site, leading to changes in the optimal pH value. We studied the thermal stability of these mutants by comparing the activities of wild‐type and mutant laccases after they were incubated for 4 h in a water bath at 80 °C (Fig. 2E,F). Similar thermal stability was observed for wild‐type lacTT, M460F and M460Q mutants, and the residual enzymatic activities were about 65%. The M460A mutant had a higher thermal stability than the wild‐type protein; it retained 80% activity. About 40% of enzymatic activity remained in the M460L and M460H mutants, displaying a reduced thermal stability.

### UV–visible spectra of wild‐type and mutant enzymes

**Figure 3:**
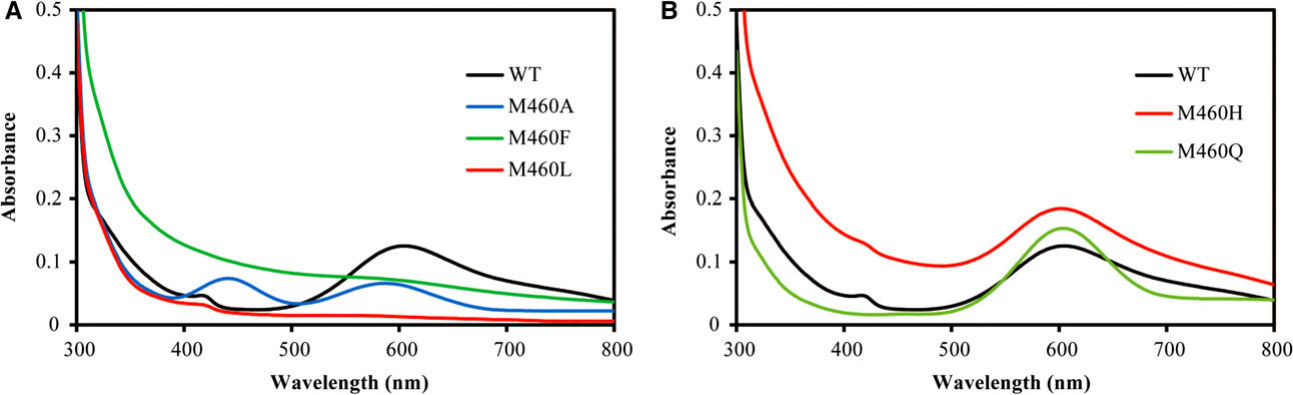
UV–visible absorption spectra of wild‐type and mutant proteins were measured at room temperature in 10 mm sodium phosphate buffer, pH 6.0. The protein concentration was 3 mg·mL^−1^. (A) WT (black), M460L (red), M460F (green) and M460A (blue); (B) WT (black), M460H (red) and M460Q (green).

In the UV–visible spectrum of wild‐type laccase, two absorption bands were observed, as shown in Fig. 3. The S_(cys450)_ π – ${\text{Cu}}_{\left(T1\right)}^{2+}$ charge transfer transition caused the intense absorption band at 608 nm 24. The broad absorption band at about 330 nm corresponded to the T3 Cu‐OH^−^ charge transfer transition 25. In the UV–visible spectra of the M460A, M460L and M460F mutants, the intensity of the absorption peak at 608 nm was lower than that of the wild‐type lacTT, indicating that the T1 Cu^2+^ population in mutants was lower. It was possible that the reduction of the T1 Cu^2+^ ion led to the decrease of the intensity of the absorption peak at 608 nm. We used potassium bichromate to treat these mutants; the intensity of the absorption band at 608 nm did not change. Thus, this possibility was ruled out. The T1 Cu^2+^ population in the M460L mutant was the most affected, with 10% of that in the wild‐type enzyme; the M460A and M460F mutants had 51% and 57% of the T1 Cu^2+^ population of wild‐type laccase, respectively (Fig. 3A and Table 2). It was also noticed that an additional absorption band appeared at about 440 nm for the M460A mutant (Fig. 3A), corresponding to a S_(Cys450)_ σ → ${\text{Cu}}_{\left(T1\right)}^{2+}$ charge transfer transition caused by a highly distorted tetrahedral geometry at the T1 Cu site 24. The absorption at 440 nm was so strong relative to that at about 608 nm that this mutant might display a green color, as observed for the purified M460A mutant protein. A small peak was also observed in UV–visible spectra of wild‐type and M460L mutant laccases. In the wild‐type, it was attributed to the S_(Met460)_ σ → ${\text{Cu}}_{\left(T1\right)}^{2+}$ charge transfer transition; in the M460L mutant, it was from the S_(Cys450)_ σ → ${\text{Cu}}_{\left(T1\right)}^{2+}$ charge transfer transition 26.

**Table 2:** The relative absorption intensity for wild‐type and mutant laccases

*A* (nm)	LacTT	M460A	M460F	M460L	M460H	M460Q
608	100%	51%	57%	10%	148%	122%
330	100%	90%	188%	87%	211%	59%

For the M460H and M460Q variants, the maximum intensities of the absorption at 608 nm were 148% and 120% of that of the wild‐type enzyme, respectively (Fig. 3B and Table 2); therefore, the M460H and M460Q variants had a higher T1 Cu^2+^ population. A small peak at 420 nm was also observed in the UV–visible spectrum of the M460H mutant, indicating that the geometry of the T1 Cu site in the M460H mutant was slightly distorted.

### Structural characteristics of wild‐type and mutant laccases

**Figure 4:**
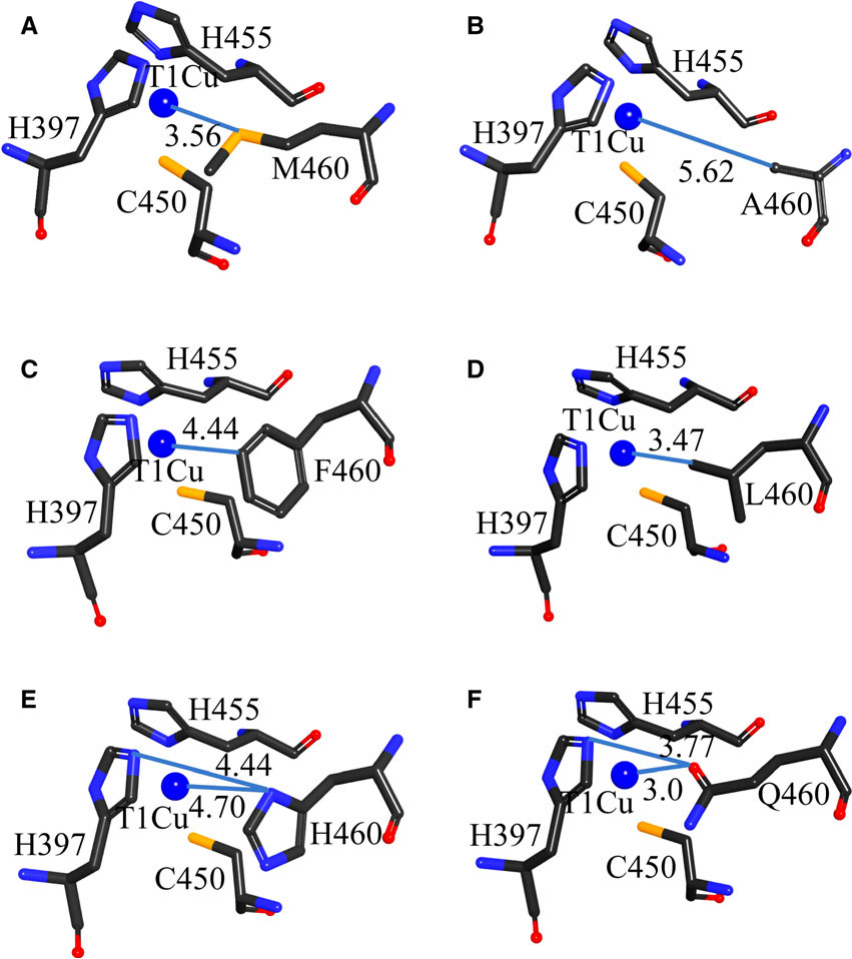
(A–D) The structural geometry of T1 Cu site for laccases WT (A), M460A (B), M460F (C) and M460L (D). The blue line denotes the distance between the T1 Cu atom and the side chain of the axial residue. (E) M460H; (F) M460Q. The blue lines indicate the distance between the T1 Cu atom and the residue at the axial position as well as the distance between the ligand His397 of T1 Cu and the axial residue. The blue ball represents the T1 Cu atom.

In order to further understand the UV–visible spectra of wild‐type and mutant proteins, we analyzed their structures generated from discovery studio 3.5. The structural model of lacTT has similar folding to other MCOs 22. It contains three domains and four Cu ions classified into two active centers (T1 Cu site and TNC), as shown in Fig. 1A. The TNC is composed of T2 Cu and two T3 Cu (T3α Cu and T3β Cu). The T2 Cu ion is coordinated by His95 and His400 residues, the T3α Cu by His137, His402 and His449 residues, and the T3β Cu by His97, His135, His451 residues. The T1 Cu ion is tetracoordinated. The His397, His455 and Cys450 residues lie on a trigonal plane and interact with the T1 Cu ion by the imidazole and thiolate moieties, respectively. At the axial position, the fourth ligand, Met460, provides the sulfur to form a weak bond with the T1 Cu ion, resulting in the tetrahedral geometry. The distance between the M460 residue and the T1 Cu atom is 3.56 Å, shown in Fig. 4A. When the Met460 residue was replaced by leucine, the side chain of the L460 residue orientated toward the T1 Cu ion, with the distance of 3.47 Å from the C^δ1^ atom to Cu and the C^δ2^ atom pointing away from the T1 Cu ion, as observed in the laccases with a leucine at the axial position 20. The geometry of the T1 Cu site in the M460L variant was retained (Fig. 4B). However, the weak van der Waals interaction between the side chain C^δ1^ of the L460 residue and the T1 Cu resulted in the low population of the Cu^2+^ ion (0.1 of the wild‐type enzyme, Fig. 3A and Table 2). In the M460F mutant, the distance between the side chain C^ε1^ of phenylalanine and T1 Cu increased to 4.44 Å in relation to the wild‐type (Fig. 4C). A weak cation‐π interaction between the copper ion and phenyl side chain maintained the geometry of the T1 Cu site but reduced the T1 Cu^2+^ population. The alanine side chain in the M460A mutant oriented in the same direction as methionine in the wild‐type, but there was a longer distance of 5.62 Å from alanine to copper as compared with the wild‐type (Fig. 4D). There was more space and no interaction between alanine and copper in the M460A mutant, which might lead to a distorted geometry and decreasing Cu^2+^ population of the T1 Cu site, consistent with the observations from UV–visible spectra.

The structural model of the M460H mutant showed that the side chain N^δ^ of His460 was 4.7 Å away from the T1 Cu ion, forming a stronger bond interaction than the S_(Met)_–Cu_(T1)_ bond in the wild‐type laccase, resulting in the increasing T1 Cu^2+^ population, and 4.4 Å away from the side chain N^ε^ of His397, a ligand of the T1 Cu ion, forming an interaction network with T1 Cu ion (Fig. 4E). These interactions could cause a slightly distorted geometry at the T1 Cu site.

### The replacement effect of the axial residue by non‐coordinating residues on the redox potential and catalytic efficiency of lacTT

The substrate is oxidized at the T1 Cu site 7. The redox potential of the T1 Cu site is a key determinant for substrate specificity and varies significantly amongst the different members of the MCOs 9. The hydrophobic environment plays an important role in modulating the redox potential of the T1 Cu site, the stronger the hydrophobic environment around the T1 Cu atom, the higher the redox potential of the T1 Cu site 16, 27. In the present study, a reducing substrate, guaiacol, was used to identify the catalytic properties of wild‐type and mutant laccases. The catalytic activities of wild‐type and M460A, M460L and M460F mutant laccases were compared at their optimum pH. The M460A and M460F mutants displayed a similar catalytic efficiency (*k*_cat_/*K*_m_) to the wild‐type enzyme, and the M460L mutant had about 5‐fold lower efficiency than the wild‐type enzyme, as shown in Table 3. UV–visible spectra revealed that the M460A, M460F and M460L mutants had a lower T1 Cu^2+^ population, 0.51, 0.57, 0.1 of that of the wild‐type enzyme, respectively (Fig. 3A and Table 2), which was reduced from the effective concentration of the enzyme; thus, the catalytic efficiency of the M460A, M460F and M460L mutants was adjusted to 1.7‐, 1.7‐ and 2.3‐fold higher than that of wild‐type enzyme, respectively. The replacement of the axial residue by non‐coordinating residues caused the increase of efficiency and decrease of *K*_m_ values; the higher efficiency for mutants most probably resulted from an increase of the substrate affinity (Table 3).

**Table 3:** Kinetic parameters of the wild‐type and mutants expressed in *Escherichia coli*. The catalytic efficiency of the enzyme is defined by *k*_cat_/*K*_m_. The values are means ± standard deviation

Protein	*K*_m_ (μm)	*k*_cat_ (s^−1^)	*k*_cat_/*K*_m_ (s^−1^·mm ^−1^)
TT	378.49 ± 18.19	5.98 ± 0.32	15.85 ± 1.63
M460A	271.84 ± 13.21	3.74 ± 0.11	13.78 ± 0.37
M460F	132.37 ± 5.06	2.07 ± 0.06	15.63 ± 1.03
M460L	72.40 ± 13.31	0.26 ± 0.01	3.69 ± 0.74
M460H	88.74 ± 7.39	3.35 × 10^−3^ ± 2.09 × 10^−4^	3.81 × 10^−2^ ± 5.27 × 10^−3^
M460Q	436.06 ± 48.28	3.18 ± 0.20	7.39 ± 0.97

The redox potentials of the T1 Cu site for wild‐type, M460A and M460F mutant laccases were determined to be 357, 409 and 416 mV respectively (Table 4). The redox potential of the M460L mutant could not be measured because it would precipitate at the concentration required for the experiment. The replacement of the axial methionine residue in the lacTT by alanine or phenylalanine resulted in an increase of the redox potential by around 50 and 60 mV, respectively. In the lacTT, the replacement of the axial methionine by an Ala or Phe residue could alter the ligand–metal interaction, which might destabilize the reduction state of the Cu atom, and the redox potential of the T1 Cu site increased 16. However, the enhancement of redox potentials did not lead to the increase in the *k*_cat_ values for mutants, indicating the redox potential was not the key factor affecting the turnover rates in these mutants.

**Table 4:** Redox potentials of wild‐type and mutant proteins

Protein	*E*° (mV)
WT	357
M460A	409
M460F	416
M460H	371
M460Q	258

### The replacement effect of the axial residue by strongly coordinating residues on the redox potential and catalytic efficiency of lacTT

The axial methionine residue was replaced by histidine or glutamine in order to further explore how an increase of the interaction between the axial residue and the copper ion affected the enzymatic behavior. Kinetic data indicated that the M460H mutant displayed 400‐fold lower efficiency and an increasing affinity of the substrate indicated by a reduced *K*_m_ value, as compared with the wild‐type. Thus, the decrease of catalytic efficiency might result from the decrease of rate constant *k*_cat_, shown in Table 3. The potential experiment indicated that the M460H mutant had 10 mV higher potential than the wild‐type laccase. A higher potential and effective concentration (148% of the wild‐type enzyme, Fig. 3B and Table 2) of the M460H mutant did not lead to the increased *k*_cat_ value. Spectral and structural analyses revealed that the interacting network formed by His397, H460 and the T1 Cu ion (Figs 3B and 4E) might lower the rate of electron transfer from T1 Cu to TNC, and further, the efficiency of the M460H mutant.

The mutation of Met460 to Gln460 led to one‐fold higher *K*_m_, two‐fold lower *k*_cat_ values, and about three‐fold lower efficiency in relation to the wild‐type laccase (Table 3), considering that the T1 Cu^2+^ population of the M460Q mutant was 20% higher than that of the wild‐type enzyme. The replacement of Met460 by glutamine shortened the distance of the ligand–metal bond from 3.56 to 3.0 Å (Fig. 4A,F), which was longer than that in other laccase with the axial residue glutamine (2.21 Å) 28. The O^ε^ atom of Gln460 could form the hydrogen‐bond with the N^ε^ atom of the ligand His397 (3.77 Å). These interactions might compress the substrate‐binding pocket, further reduced the substrate affinity in the M460Q mutant, consistent with the higher *K*_m_ value of the M460Q variant. The redox potential of M460Q mutant was determined to be 258 mV, about 100 mV lower than that of the wild‐type laccase (Table 4), which also contributed to the decrease of the *k*_cat_ value.

## Discussion

A wide range of studies have been reported for laccases from different sources. These laccase have highly conservative advanced structures, but a low homology in amino acid sequences 29, 30. The residue at the axial position is non‐conserved. Leucine, phenylalanine or methionine is found as the axial residue in different organisms 12. Site‐directed mutagenesis of the axial residue, such as Leu/Met mutant of the laccase from *Botrytis aclada* 20, Met/Leu and Met/Phe mutants of the laccase from *Bacillus subtilis* 16 and the replacement of axial Phe463 in *Trametes villosa* laccase by residue Leu or Met 12 demonstrated that the mutant laccases with phenylalanine or leucine as the axial residue exhibited higher redox potentials than or equal to those of the mutants with the axial methionine. In the lacTT, the T1 copper ion is located in a geometry formed by two histidines, one cysteine and one axial amino acid residue (Fig. 1B). The distance between the S^δ^ atom of the axial methionine and the T1 Cu atom is 3.56 Å, as in the laccase from *T. thermophilus* HB27 with a methionine at the axial position 31. The redox potentials of mutants with hydrophobic axial residues (Ala and Phe) were higher than that of the lacTT, consistent with previous reports 16. The structural comparison of the M460F mutant with wild‐type enzyme displayed no changes except for the mutation site nearby; these findings were also observed in the L499M mutant from *Botrytis aclada* laccase and M502F mutant from *Bacillus subtilis* laccase 16, 20. However, there were the gradually enhanced interactions between the T1 Cu^2+^ and axial ligand for wild‐type and mutant lacTT, a weaker cation‐π interaction between the phenyl ring of phenylalanine and the T1 Cu^2+^ ion in the M460F mutant, a Cu–S bond interaction in the lacTT, strong N^δ^_His460_–Cu_T1_ and stronger O^ε^_Gln460_–Cu_T1_ bonds in the M460H and M460Q mutants respectively. The redox potentials of laccases decreased with the increase of the axial ligand–metal interaction (Table 4). Thus, the alteration of the redox potential was most probably ascribed to the change in the type of the axial residue 16, 32, 33. However, the M460A mutant without axial ligand–metal interaction displayed a smaller redox potential than the M460F variant, probably due to the highly distorted geometry of the T1 Cu site. Apart from the chemical nature of the axial residue, there are other factors that affect the redox potential of the laccase, such as hydrogen‐bond networks around the copper, solvent accessibility of the T1 Cu site and charge dipole interaction between the backbone and the metal site 34. For *B. subtilis* laccase, the substitution of leucine at the second coordination sphere resulted in a 60 mV decrease in the redox potential 6. In addition, the metal–ligand interaction might play a role in the stability of the T1 Cu^2+^ ion, as indicated by the UV–visible spectra.

If the rate‐limiting step of laccase catalysis is the oxidation of substrate at the T1 Cu site, it is most likely controlled by the redox potential difference between this site and the substrate 13, 23. The mutants with higher redox potentials than the wild‐type are expected to display higher activities. However, in this study, the M460A and M460F mutants with higher redox potentials displayed reduced turnover number (*k*_cat_) in the oxidation of guaiacol (Table 3). Thus, the redox potential difference between the T1 Cu and the substrate was not rate‐limiting, because the guaiacol has a relatively low redox potential and is easily oxidized 35. In addition, the replacement at the axial position of T1 Cu ion might disturb the intramolecular electron transfer pathway from the T1 Cu to the TNC as indicated by the alterations in the intensity of absorption peaks at 330 nm (Fig. 3) further reducing the electron transfer rate. Therefore, the *k*_cat_ value of the reaction decreased.

From this study, the catalytic efficiency of mutant proteins with hydrophobic, non‐coordinating axial residues (alanine, phenylalanine or leucine) increased by different extents, but these mutants showed a reduced effective concentration. Mutants with hydrophilic, strongly coordinating axial residues (glutamine or histidine) displayed reduced catalytic efficiency as compared with the wild‐type laccase. These results suggested that it might be impossible to obtain mutants with enhanced behavior by the mutation at the axial ligand, but these results might provide some insight into the catalytic mechanism of laccase, beneficial to fine‐tune enzymatic properties for the industrial application by protein engineering techniques. For example, a substitution distant from the T1 Cu ion might cause an increase in redox potential of the copper center, simultaneously with less severe disturbance in the intramolecular electron transfer pathway, so the conversion rate could be expected to increase, as observed in the laccase from *Streptomyces sviceus* 36.

## Conflict of interest

The authors declare no conflict of interest.

## Author contributions

Y. Zhu acquired and analyzed the data and wrote the paper, Y. Zhang and JZ acquired the data, YL provided helpful suggestions during the experiments and the paper‐writing, and XY conceived and designed the project, analyzed and interpreted the data and wrote the paper.
